# Molecular-enriched functional connectivity in the human brain using multiband multi-echo simultaneous ASL/BOLD fMRI

**DOI:** 10.1038/s41598-023-38573-0

**Published:** 2023-07-20

**Authors:** Ottavia Dipasquale, Alexander Cohen, Daniel Martins, Fernando Zelaya, Federico Turkheimer, Mattia Veronese, Mitul A. Mehta, Steven C. R. Williams, Baolian Yang, Suchandrima Banerjee, Yang Wang

**Affiliations:** 1 Department of Neuroimaging, Institute of Psychiatry, Psychology and Neuroscience, King’s College London, De Crespigny Park, London, SE5 8AF UK; 2 Department of Radiology, Medical College of Wisconsin, 8701 Watertown Plank Road, Milwaukee, WI 53226 USA; 3 Department of Information Engineering, University of Padova, Padua, Italy; 4 GE Healthcare, Waukesha, WI USA; 5 GE Healthcare, Menlo Park, CA USA

**Keywords:** Neural circuits, Molecular neuroscience

## Abstract

Receptor-enriched analysis of functional connectivity by targets (REACT) is a strategy to enrich functional MRI (fMRI) data with molecular information on the neurotransmitter distribution density in the human brain, providing a biological basis to the functional connectivity (FC) analysis. Although this approach has been used in BOLD fMRI studies only so far, extending its use to ASL imaging would provide many advantages, including the more direct link of ASL with neuronal activity compared to BOLD and its suitability for pharmacological MRI studies assessing drug effects on baseline brain function. Here, we applied REACT to simultaneous ASL/BOLD resting-state fMRI data of 29 healthy subjects and estimated the ASL and BOLD FC maps related to six molecular systems. We then compared the ASL and BOLD FC maps in terms of spatial similarity, and evaluated and compared the test–retest reproducibility of each modality. We found robust spatial patterns of molecular-enriched FC for both modalities, moderate similarity between BOLD and ASL FC maps and comparable reproducibility for all but one molecular-enriched functional networks. Our findings showed that ASL is as informative as BOLD in detecting functional circuits associated with specific molecular pathways, and that the two modalities may provide complementary information related to these circuits.

## Introduction

Understanding the relationship between the microscale molecular processes of the human brain and its macroscale functional architecture has attracted considerable attention in the neuroimaging community over the years^1–6^. The use of multimodal approaches integrating outputs from different techniques and acquisition sequences can enrich the information that can be derived compared to single modalities when studying brain function^7^. This integration has also been fuelled by recent advances in analytical approaches^2,3^ and global data-sharing initiatives (e.g. UK Biobank, ENIGMA Consortium, Human Connectome Project, etc.).

One of the most recent analytical strategies to integrate functional magnetic resonance imaging (fMRI) and molecular imaging is the Receptor-Enriched Analysis of functional connectivity by targets (REACT)^2^, a novel multimodal method that exploits the temporal dynamics of fMRI signal to identify functional circuits associated with specific molecular systems. By using neuroimaging templates of receptor density derived from Positron Emission Tomography (PET) or Single-Photon Emission Computerized Tomography (SPECT) imaging in healthy subjects, REACT maps fMRI data onto the space of specific molecular systems and recovers biologically informed networks of functional connectivity (FC).

The application of REACT to clinical and pharmacological blood oxygen level-dependent (BOLD) fMRI studies has demonstrated its ability to uncover new patterns of functional alterations in neurotransmission-related circuits in patients with brain disorders and suggests potential drug-target mechanisms at the system level that could underlie the functional effects of pharmacological treatment^2,8–12^. However, BOLD signal is an indirect measure of neuronal activity, resulting from the combination of changes in both oxygen consumption and regional cerebral blood flow (CBF), as well as changes in regional blood volume (CBV)^13^. Thus, inferring neuronal dynamics directly from the BOLD signal is not straightforward.

Arterial Spin Labelling (ASL) offers an exciting alternative to BOLD since it allows for a more specific measure of global and regional CBF changes associated with neuronal^14–16^ and neurotransmitter activity^17,18^. CBF is also suitable for pharmacological MRI studies assessing drug effects on baseline brain function^19^, it is acquired with pulse sequences that are resistant to magnetic field inhomogeneity effects^20^, thus avoiding susceptibility artifacts and signal loss in the orbitofrontal, inferior temporal, and limbic regions that are linked to major neurotransmitter systems^21^, and its signal is typically well localized to the capillary beds^22,23^. However, some of the classical technical limitations of the standard single-shot ASL acquisition techniques (e.g., long tagging time to label inflowing blood and post-labeling delay to allow tagged blood to flow into the brain, which inevitably result in a lower spatial and temporal resolution compared to BOLD imaging) have hampered the use of ASL as a reliable measure of brain FC.

A novel multi-band and multi-echo (MB-ME) ASL/BOLD sequence has been recently developed to overcome some of those limitations and provide high-resolution, whole-brain simultaneous ASL/BOLD data to extend the study of functional connectivity in the human brain^24^. This sequence exploits the advantages of MB and ME techniques to improve signal to noise ratio (SNR) and increase the temporal resolution of ASL data. The collection of more than two echoes also allows for the use of advanced data-driven de-noising strategies, including multi-echo independent component analysis (ME-ICA)^25,26^, resulting in the identification of reliable resting state networks, higher CBF/BOLD coupling, and increased signal stability and reproducibility^24,27^. Data obtained with this MB-ME ASL/BOLD sequence have already been used to estimate and compare ASL- and BOLD-based resting state networks, and to evaluate the coupling of CBF and BOLD signals^24,27^.

The above mentioned benefits of ASL, its usage as a quantitative surrogate marker of brain function and the latest technical developments of ASL fMRI offer the opportunity to integrate ASL data and molecular imaging with REACT, to gain further insights from underlying neurotransmitter density data and evaluate brain response to pharmacological intervention. If feasible, this application could pave the way to the integration of molecular and functional imaging in pharmacological and clinical studies where resting-state BOLD data were not—or are not intended to be—acquired, providing a biologically-informed tool to complement the evaluation of global and regional changes in CBF.

In this study, we determined the feasibility of using REACT with ASL fMRI data by estimating the ASL-based FC networks related to six molecular systems that have been used in previous BOLD-based REACT studies, and compared the resulting ASL-based molecular-enriched functional networks with those derived from BOLD fMRI data in terms of spatial configuration and test–retest reliability. By using ASL and BOLD data acquired simultaneously rather than sequentially, we were able to perform an unbiased comparison of the two modalities and identify the intrinsic differences between them, ruling out the possibility that any spatial differences in the ASL- and BOLD-derived networks might be induced by physiological confounds (e.g., different levels of alertness could potentially affect the spatial pattern of the noradrenergic network).

## Methods

### Participants and MRI data acquisition

Twenty-nine right-handed healthy volunteers (mean age = 28.0 years, age range 20–46, males/females: 9/20) were recruited for this study. Nineteen of them returned within 2 weeks to repeat the study. Subjects were instructed to refrain from caffeine and tobacco for 6 h prior to the MRI exam.

All subjects provided written informed consent prior to participation in this study, which was approved by the Medical College of Wisconsin Institutional Review Board and conducted in accordance with the Declaration of Helsinki. All methods were carried out in accordance with the approved guidelines and regulations.

Imaging was performed on a 3 T MR scanner (Signa Premier, GE Healthcare, Waukesha, WI, United States) with a body transmit coil and a 32-channel NOVA (Nova Medical, Wilmington, MA, United States) receive head coil. Subjects underwent a resting state fMRI scan using a MB-ME PCASL/BOLD EPI sequence^24,27,28^ with the following acquisition parameters: echo times (TEs)/repetition time (TR) = 11,30,48,67/3500 ms, FOV = 24 cm, matrix size = 80 × 80, slice thickness = 3 mm (3 × 3x3 mm voxel size), 11 excitations with multiband factor = 4 (44 slices in total), FA = 90°, partial Fourier factor = 0.85, and in-plane acceleration (R) = 2, number of time points = 96, total scan duration = 5.6 min. PCASL parameters included labelling time = 1450 ms and post labelling delay time (PLD) = 1000 ms. One full label/control pair was acquired every 7000 ms. Subjects were instructed to lie still with their eyes closed and stay awake.

A 3D T1-weighted magnetization-prepared rapid acquisition with gradient echo (MPRAGE) anatomical image was also collected to aid with coregistration (TR/TE = 2200/2.8 ms, field of view (FOV) = 24 cm, matrix size = 512 × 512 × 256, slice thickness = 0.5 mm, voxel size = 0.47 × 0.47 × 0.5 mm, and flip angle (FA) = 8°).

### Image pre-processing

Data was analyzed using a combination of AFNI^29^ and FSL^30^. First, the anatomical MPRAGE image was coregistered to Montreal Neurological Institute (MNI) space. Each fMRI dataset was then volume registered to the first volume using *mcflirt* in FSL. Importantly, only the first-echo dataset was registered, and both label and control images were included. Subsequent echoes were registered using the transformation matrices from the first echo.

For the BOLD analysis, the four echoes were combined using the ${\mathrm{T}}_{2}^{*}$-weighted approach^31^. The data was then denoised using ME-ICA and the open source python script tedana.py version 0.0.10 (https://tedana.readthedocs.io/en/latest)^25,26,32^ to reduce mainly motion-related artefacts, but also other non-BOLD sources of noise^25,33^. This technique, described in detail elsewhere, classifies independent components as BOLD or non-BOLD based on whether or not their amplitudes are linearly dependent on TE, respectively^25,26,34^, and regresses non-BOLD components out of the combined ME data, returning a denoised MBME dataset. Because both label and control volumes were included as input into tedana, in all cases a label-control oscillation noise component was identified and regressed from the BOLD data^24^. The denoised dataset was then registered to the MPRAGE image using *epi_reg* in FSL and then to MNI space using the anatomical transformations computed previously. The data was then smoothed using a 6 mm FWHM Gaussian kernel, and high-pass filtered with f_c_ = 0.01 Hz. Finally, to remove residual unspecific confounders (e.g., scanner instabilities, physiological drifts, cardiac fluctuations and respiration)^35–38^, we applied a standard denoising step for BOLD data based on the regression of the white matter (WM) and cerebrospinal fluid (CSF) signals. To perform this step, we first created group masks of the WM and CSF from the MNI152 standard template and used those masks to extract the mean WM and CSF signals from the BOLD fMRI data. WM and CSF signals were then regressed out of the BOLD data using *fsl_glm* in FSL.

For the ASL data analysis, the first-echo data was registered to the MPRAGE image and then to MNI space using the anatomical transformations computed previously. The data was then smoothed using a 6 mm FWHM Gaussian kernel, and a perfusion-weighted (PW) time series was computed by surround subtracting label and control images^14^. In order to improve spatial specificity and increase SNR by reducing interindividual physiological differences in ASL data^39^, we regressed the global PW signal out of the ASL data. To perform this step, we estimated for each time point the mean PW value across the grey matter voxels^40,41^ and regressed the signal out of the ASL dataset using the *fsl_glm* function in FSL.

### Molecular-enriched functional connectivity analysis with REACT

Six publicly available in vivo molecular templates of the major neuromodulatory systems, including the transporters of dopamine (DAT)^42^, noradrenaline (NAT)^43^, serotonin (SERT)^44^ and vesicular acetylcholine (VAChT)^45^, and GABA-A and mGlu5 receptors^46,47^ were used as spatial priors to enrich the fMRI analysis of the BOLD and ASL data and estimate the corresponding functional circuits related to each molecular system. We selected these templates to provide illustrative examples of the methodology and its general usage with ASL and BOLD data, although different types of molecular templates can be used in REACT, according to the specific hypotheses of the study and the template availability (e.g., to explore the brain mechanisms underlying a certain disorder, neurotransmitter transporters might be more suited to capture the full architecture of a certain system, while in a drug challenge targeting specific neurotransmitters, the optimal approach would be to use the maps of those specific receptors, if drug binding is known^48^). Every molecular template was previously normalized by scaling its image values between 0 and 1 and masked using a standard grey matter mask. Of note, the regions used as references for quantification of the molecular data in the kinetic models for the radioligands were masked out of the corresponding template, namely the occipital areas in DAT and NAT and the cerebellum in SERT, VAChT, and mGluR5. The original templates can be found at https://github.com/netneurolab/neuromaps^49,50^. To examine collinearity between the receptor systems, we calculated the correlation coefficients between each pair of molecular templates as well as their Variance Inflation Factors (VIFs). Of note, VIF quantifies the severity of multicollinearity in an ordinary least squares regression analysis (VIF = $\frac{1}{1-{R}^{2}}$). VIFs higher than 1 and up to 5 typically suggest that there is a moderate correlation between two variables, but it is not severe enough to warrant corrective measures, while values greater than 5 represent critical levels of multicollinearity and will result in a poor estimation of the coefficients, meaning that alternative analytic approaches (e.g., dimensionality reduction) are required^51–53^.

A detailed explanation of the REACT methodology and its applications can be found elsewhere^2,10^. In brief, the functional circuits related to the six molecular systems were estimated using a two-step multiple regression analysis. In the first step, the fMRI volumes were masked using a binarized mask derived from the molecular templates to restrict the analysis to the voxels for which the density information of the neurotransmitters was available. Then, the molecular templates were used as a set of spatial regressors to weight the fMRI images and estimate the dominant fluctuation related to each molecular system at the subject level. The resulting subject-specific time series were then used as temporal regressors in a second multiple regression analysis to estimate the subject-specific spatial map associated with each molecular template. The output consists of six maps per subject and fMRI dataset, each one reflecting the molecular-enriched FC associated with a specific neurotransmitter. This analysis was performed using the react-fmri package (https://github.com/ottaviadipasquale/react-fmri)^54^.

### Comparison of BOLD and ASL-based molecular-enriched FC

To estimate population inferences for each neuroreceptor system and fMRI dataset (BOLD and ASL), we performed voxel-wise one-sample t-tests using *randomise* in FSL^55^ (one test for positive FC and one for negative FC). Of note, t-tests in *randomise* are always one-tailed. To make them two-tailed, one needs to run the two contrasts separately and then correct for multiple comparisons across contrasts.

The resulting t-stat images of positive and negative FC were compared between fMRI datasets using the Dice Similarity Index as well as the voxel-wise spatial correlation, to assess the spatial similarity between ASL- and BOLD-derived molecular-enriched FC maps.

#### Dice similarity index (DSI)

After thresholding the t-stat images at p_FWE_ < 0.05 (corrected for multiple comparisons across voxels using the threshold-free cluster enhancement (TFCE) option^56^, and across contrasts using Bonferroni correction), and binarizing them, we measured the degree of overlap between the different fMRI datasets using the DSI, calculated as:

$$\mathrm{DSI}=\frac{2|\mathrm{X}\cap \mathrm{Y}|}{\left|\mathrm{X}\right|+|\mathrm{Y}|},$$

where X are all active voxels in the BOLD-derived FC maps and Y are all active voxels in the ASL-derived FC map. To measure the total DSI, we first counted the voxels with concordant FC in the two modalities (i.e., X^+^ ∩ Y^+^ and X^−^ ∩ Y^−^), and then added them together to obtain a single |X ∩ Y| term. The DSI denominator was calculated considering the total (positive + negative) FC voxels for both modalities. The comparison between BOLD and ASL FC was also run separately for the positive and negative FC (DSI^+^ and DSI^−^, respectively).

#### Voxel-wise spatial correlation

To investigate the linear relationship of the within-group FC patterns between modalities, for each molecular-enriched functional network we computed the Pearson’s correlation coefficient between the unthresholded BOLD and ASL t-stat maps resulting from the one-sample t-tests. This estimation was performed using a parameterized generative method implemented in the brainSMASH platform^57^ to account for the inherent spatial autocorrelation of the data. Of note, the t-stat maps were down-sampled at 4 mm^3^ to reduce the computational burden of the calculations.

### Test–retest analysis

The reproducibility of BOLD and ASL response in generating consistent molecular-enriched functional networks was quantified using the regional intraclass correlation (ICC) between test and retest sessions. First, for each molecular-enriched functional network, dataset (BOLD and ASL) and subject having test and retest data (n = 19), we parcellated the FC map using the Desikan-Killiany atlas and estimated the mean FC of each region. Then, we calculated the regional ICC between the test and retest sessions for each fMRI dataset, ROI and network:

$$\mathrm{ICC}\left(\mathrm{3,1}\right)=\frac{{MS}_{B}-{MS}_{E}}{{MS}_{B}+\left(\mathrm{K}-1\right){MS}_{E}},$$

where MS_B_ is the mean square variance between subjects and MS_E_ the mean square error between sessions. MS_B_ and MS_E_ were calculated from an n × k matrix with n = 19 observations and k = 2 measurements. This resulted in one ICC value per fMRI dataset and ROI of each network.

In order to compare the reproducibility of the two modalities, the resulting ICC values of each network were Fisher’s z transformed and compared between BOLD and ASL datasets using non-parametric Wilcoxon signed-rank paired sample t-tests, after testing for normality.

## Results

The VIF values for DAT, NAT, SERT, VAChT, GABA and mGlu5 maps were 1.8898, 1.6989, 3.3826, 3.4414, 2.5160 and 2.9742 respectively, reflecting a low to moderate level of collinearity and confirming their suitability for inclusion together within the multiple linear regression analysis of REACT.

**Figure 1:**
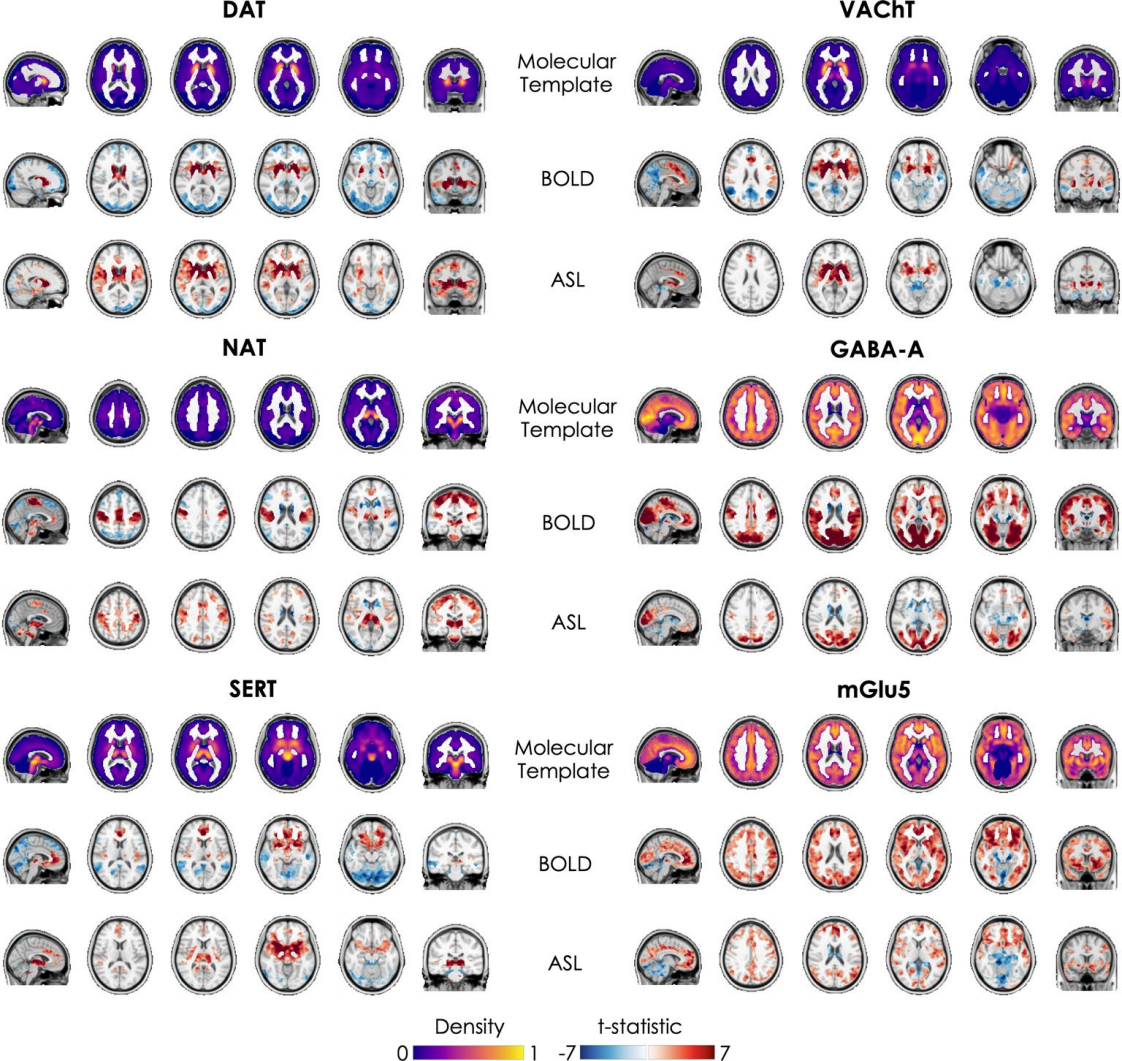
Molecular templates and corresponding molecular-enriched FC maps derived from BOLD and ASL datasets. The distribution density of the molecular templates was rescaled between 0 and 1 and the regions used as references in the kinetic models were set at 0. The molecular-enriched FC maps are obtained from the significant t-stat maps resulting from the one sample t-tests performed on the positive FC (in red) and negative FC (in blue) (p_FWE_ < 0.05, corrected for multiple comparisons across voxels using the TFCE option and contrasts with Bonferroni correction).

Figure 1 shows the normalized molecular templates of DAT, NAT, SERT, VAChT, and GABA-A and mGlu5 receptors, and the corresponding significant t-stat BOLD- and ASL-derived FC maps resulting from the one sample t-tests, thresholded at p_FWE_ < 0.05 corrected for multiple comparisons across voxels using the TFCE option^56^ and contrasts (one for positive FC and one for negative FC) using Bonferroni correction.

Overall, the two fMRI datasets show good consistency in terms of identification of the main hubs (i.e., regions with positive FC indicating their association with the functional network) of each molecular-enriched network, which align with the distribution of their molecular systems and related pathways. The main hub of the DAT-enriched network corresponds to the striatum (i.e., caudate and putamen), although it is also possible to identify other key cortical and subcortical regions as belonging to this network, including the thalamus, anterior cingulate gyrus, paracingulate gyrus, insula, superior frontal gyrus, opercular cortex, precentral gyrus, superior temporal gyrus and temporal pole. The key regions of the NAT-enriched network identified by both fMRI datasets are the brainstem, thalamus, pre- and postcentral gyri, supplementary motor cortex, insula and part of the anterior cingulate cortex, as previously reported in other studies^8,10^. The SERT-enriched network is mainly localized in the thalamus, putamen, midbrain, insula, anterior cingulate gyrus, frontal orbital cortex, paracingulate gyrus, subcallosal cortex and frontal pole. Both datasets identified the striatum, insula, anterior cingulate gyrus, paracingulate gyrus and precentral gyrus as key regions of the VAChT-enriched network. The functional networks related to GABA-A and mGlu5 receptors emerged as more widespread across cortical regions, in line with their wider expression throughout the cortex. The areas belonging to the GABA-A-enriched network identified by both datasets are the occipital pole and lateral occipital cortex, lingual gyrus, intracalcarine cortex, cuneus and precuneous, pre- and postcentral gyri, occipital fusiform gyrus and superior parietal lobule, while the mGlu5-enriched network was mainly localized in the frontal pole, frontal orbital cortex, superior and middle frontal gyri, anterior cingulate gyrus, lateral occipital cortex, occipital pole, paracingulate gyrus, insular cortex, precuneous, angular gyrus, pre- and postcentral gyri and middle temporal gyrus. The complete list of regions with significant within-group positive FC in the molecular-enriched functional maps derived from each dataset can be found in Supplementary Fig. 1.

### Spatial similarity between datasets

**Figure 2:**
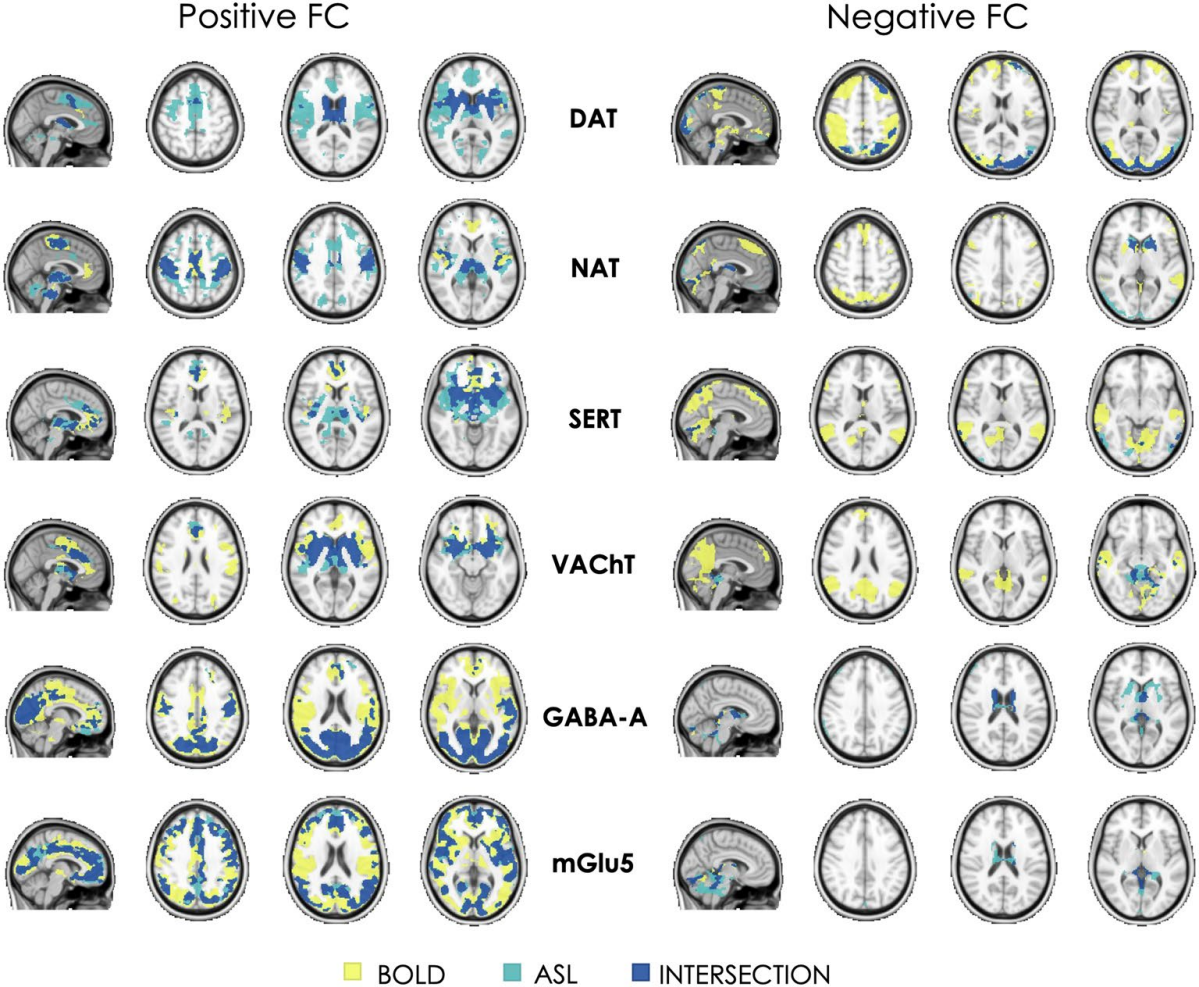
Binary representation of the spatial distribution of the molecular-enriched functional networks derived from BOLD and ASL images and their overlap. The overlap of regions with positive FC and regions with negative FC are represented in two separate panels (on the left and right, respectively).

Results from the comparison between datasets estimated using the DSI are reported in Table 1 and visually represented in Fig. 2. Overall, the BOLD- and ASL-derived maps showed a low to moderate similarity when considering positive and negative FC together (total DSI range: 0.32–0.54). However, a higher spatial similarity was generally observed for positive FC than for negative FC (DSI^+^ range: 0.29–0.55; DSI^−^ range: 0.13–0.49), with the BOLD dataset showing more widespread inter-subject consistency (i.e., significant one-sample t-test for negative FC in more areas) than the ASL dataset in the regions showing a negative coupling with the main networks.

**Table 1:** Spatial similarity of the molecular-enriched functional networks between datasets (BOLD and ASL) expressed in terms of Dice Similarity Index (DSI, range 0–1) and voxel-wise Pearson’s correlation.

	Total DSI	DSI^+^	DSI^−^	Spatial correlation
DAT	0.33	0.29	0.35	0.62
NAT	0.45	0.48	0.39	0.57
SERT	0.32	0.45	0.23	0.50
VAChT	0.34	0.49	0.13	0.58
GABA-A	0.49	0.51	0.37	0.66
mGlu5	0.54	0.55	0.49	0.65

The DSI was estimated considering positive and negative FC together (Total DSI) and separately (DSI^+^ and DSI^−^, respectively). In terms of voxel-wise Pearson’s correlation, all networks showed significant correlations between datasets (p_corr_ < 0.001).

**Figure 3:**
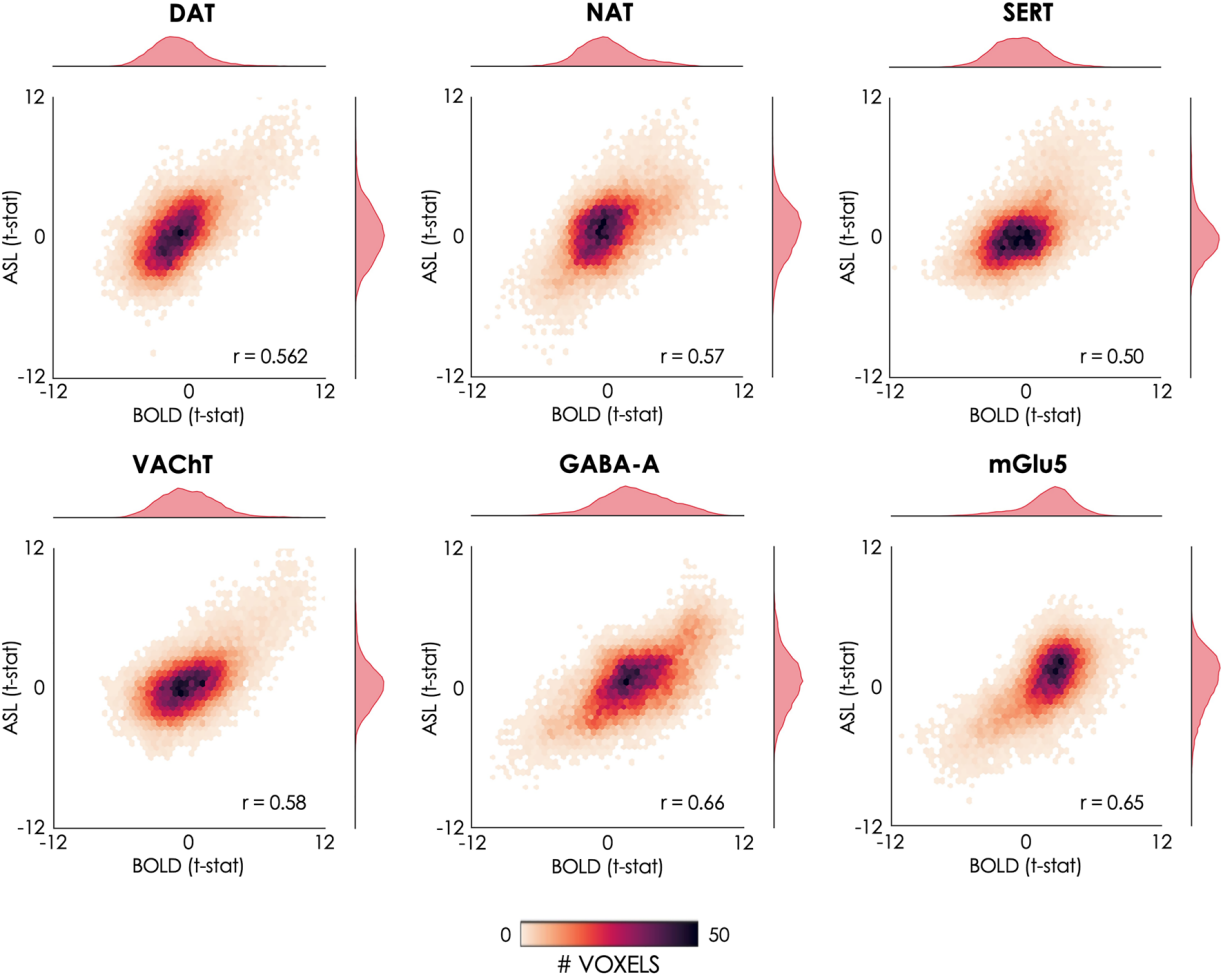
Voxel-wise spatial correlation of the molecular-enriched FC maps derived from the BOLD and ASL datasets. The 2D histograms show the relationships between the unthresholded BOLD and ASL t-stat maps resulting from the one-sample t-tests in terms of their spatial distributions across the brain. The marginal histograms on top and on the right of each 2D histogram show the modality-specific distributions of the t-stat maps.

In terms of voxel-wise spatial correlation between datasets, we found significant positive correlations between the BOLD- and ASL-derived molecular-enriched FC maps (range of Pearson’s r: 0.50–0.66, p_corr_ < 0.001). All Pearson’s correlations are reported in Table 1 and visually represented in Fig. 3.

### Results of the test–retest analysis

**Figure 4:**
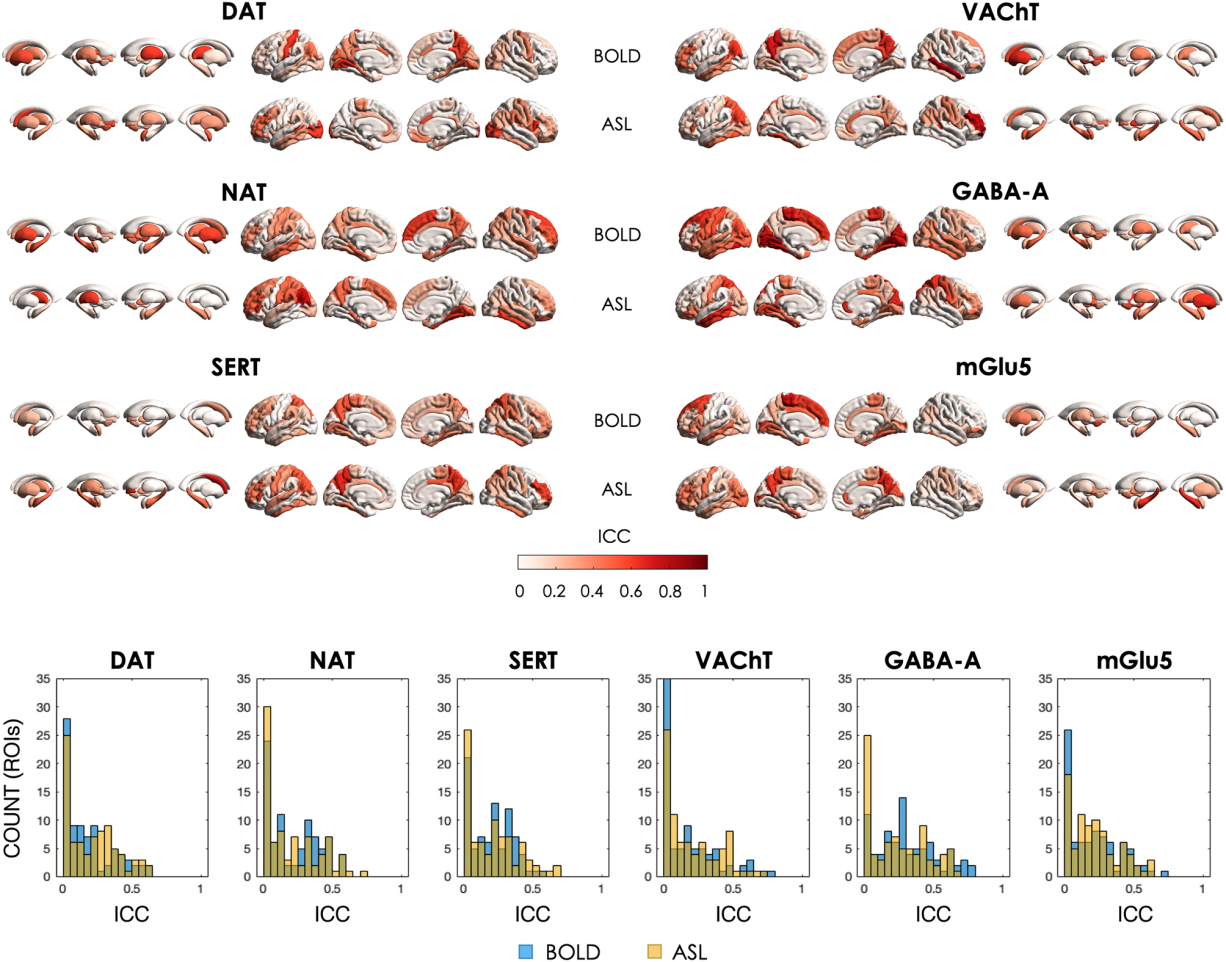
Regional Intraclass correlations (ICCs) of the test–retest sessions for each molecular-enriched functional network derived from the BOLD and ASL datasets and corresponding histograms of their distributions.

Overall, both BOLD and ASL datasets showed heterogeneity in the regional distribution of ICC across the whole brain, spanning from very low to moderate ICC (Fig. 4). As resulted from the Wilcoxon signed-rank t-tests comparing the network reliability between ASL and BOLD datasets, significant differences were found only in the regional ICCs of the GABA-A-enriched functional network (ASL < BOLD, z-score = 2.121, p < 0.034), while the other networks showed comparable reliability between modalities (ASL < BOLD, DAT: z-score = 1.253; NAT: z-score = − 0.507; SERT: z-score = 0.222; VAChT: z-score = 1.003; mGlu5: z-score = 0.503). Of note, since the statistical tests were simply applied to help quantify the comparisons between modalities without doing any further statistical inference on the difference of ICC, the results did not need correction for multiple comparisons.

## Discussion

In this study, we tested the feasibility of using ASL fMRI data to estimate molecular-enriched functional networks using REACT, a novel analytical method used to project fMRI data onto selected molecular spaces and characterize the functional coactivation of brain regions in terms of their relationship with molecular targets. Specifically, we estimated the functional networks related to six molecular systems (DAT, NAT, SERT, VAChT, GABA-A and mGlu5) using high-resolution, whole-brain resting-state ASL and BOLD fMRI data of healthy subjects acquired with a simultaneous MB-ME ASL/BOLD EPI sequence, and compared the networks resulting from the two modalities in terms of spatial similarity and test–retest reproducibility.

Similarly to the networks derived from BOLD data, the networks resulting from the ASL dataset were able to highlight, for each molecular system, both the areas of high distribution density of the neurotransmitters and their functional links with other brain areas. For example, the main regions belonging to the DAT-enriched functional network estimated from both datasets include the striatal areas, which are the most enriched in dopaminergic innervation, but also specific cortical areas implicated in dopamine modulation of reward, learning, and motivation, such as the anterior cingulate cortex^58,59^, which are functionally and anatomically connected with the striatum^60–62^. The NAT-enriched functional network shows high positive FC partly in the brainstem, which is the major site of noradrenergic neurons (mainly the locus coeruleus), but also in the thalamus and sensorimotor regions, in line with the modulatory effect of noradrenergic pathways on processing of salient sensory information via actions on sensory, attentional and motor processes^63^. Importantly, the spatial configuration of such functional networks is also comparable with the functional networks reported in previous REACT-based studies using BOLD fMRI data^2,8–10^.

### ASL versus BOLD: same but different molecular-enriched networks

Despite the lower SNR of ASL compared to BOLD imaging, our findings show that the spatial patterns of the molecular-enriched functional networks estimated from BOLD and ASL datasets overlap in many areas, as indicated by the moderate network similarity in terms of DSI and voxel-wise spatial correlation between them. These results confirm the feasibility of using ASL for the molecular-enriched fMRI analysis, but they also show that the networks identified with ASL data do not fully match the BOLD-derived ones. This finding suggests that the two modalities might capture slightly different characteristics of the functional interaction between brain regions within the molecular-enriched networks (e.g. biological differences in the dynamics of spontaneous fluctuations in CBF and BOLD), and could hence be complementary. However, we cannot fully exclude that these differences might also reflect, to some extent, intrinsic differences between modalities, such as SNR or others. For instance, since the BOLD signal originates primarily from the differences in intravascular deoxyhaemoglobin concentration, the spatial correlation to the actual site of neural activity is relatively poor with considerable spatial spreading onto the venous structures^64,65^. This phenomenon might, at least theoretically, inflate the amount of shared variance in BOLD signal fluctuations across voxels from the same venous territory. Conversely, ASL typically yields better spatial correlations with the actual site of regional involvement compared to BOLD because the signal originates from smaller calibre vessels^23^. An interesting detail that emerges from the DSI^-^ (see also the right panel of Fig. 2), is that the BOLD-derived maps have a more widespread inter-subject consistency (i.e., significant one-sample t-test in more brain regions) than the ASL ones in the regions with a negative coupling with the main networks. It must be noted that a larger extent of negative FC in BOLD networks as compared to the ASL ones has already been described in the literature^66^. These converging findings suggest that the spatial patterns of positive connectivity are more consistent across techniques than those of negative FC, which could be more dependent on the type of data (BOLD or ASL fMRI) and de-noising steps applied, if any^67^. Furthermore, it is worth noting that a widely adopted strategy to address the well-known difficulty of providing a biological meaning to negative FC, especially in graph-based studies and in works comparing BOLD- and ASL-derived FC^68,69^, is to set a FC threshold at 0 and focus on positive connectivity only.

Overall, further studies will be essential to clarify these between-modality differences and provide additional information on the spatial configuration of the BOLD and ASL networks in other contexts, such as in task fMRI or drug studies.

### Reproducibility

In terms of test–retest reproducibility, both datasets showed large variability in regional ICC across brain regions, spanning from low to good ICC (i.e., around 0.75), similarly to what has been reported in other fMRI studies^70–72^. The ASL dataset showed a significantly lower ICC than BOLD in the GABA-A-enriched network, in line with a previous study indicating that BOLD data produce more reliable spatial network patterns than ASL data^69^. On the other hand, our results showed generally lower BOLD ICC values than those reported in Ref.^69^, and comparable ICCs between ASL and BOLD in the other five molecular-enriched functional networks. This finding is in line with another study reporting comparable averaged ICCs of within-network connectivity between the two fMRI modalities^73^ and might be explained, at least in part, by the fact that the processing pipeline of our BOLD dataset, as well as the one of the just mentioned study^73^, included a step of WM and CSF signal regression to reduce the contribution of physiological noise (i.e., respiration and cardiac pulsation). This type of noise is known to generate highly reproducible, but spurious, connectivity patterns in rs-fMRI^36^, which we might have reduced by regressing WM and CSF signals from our BOLD data. Further studies expanding our approach to task-based fMRI data, where specific brain regions are expected to be engaged, could offer a more comprehensive overview of the reproducibility of these modalities in different contexts.

### Limitations

Our study has some limitations worth mentioning. First, the number of subjects was relatively small. However, the purpose of this study was to determine the feasibility of using ASL data to identify molecular-enriched networks comparable to those obtained with BOLD fMRI. Hence, the sample size was appropriate to answer our research question. Second, we explored the spatial configuration of molecular-enriched functional networks of healthy subjects at rest. Although this is the first essential step to define the feasibility of this analysis and pave the way for future works integrating molecular and ASL fMRI imaging, it is important to mention that further studies are needed in order to characterize in detail the similarities and differences between BOLD- and ASL-based molecular-enriched functional networks and investigate whether these might capture distinct aspects of the biology of the brain or simply reflect methodological differences between modalities. This could be tested using pharmacological MRI data to investigate the brain response to a drug acting on selective targets^74^, or fMRI data with simple tasks that trigger functional changes modulated by specific neurotransmitters (e.g., attentional tasks requiring modulation of the functional network related to the noradrenergic circuit). This would provide better opportunities to explore the specific characteristics of BOLD and ASL functional networks and how they respond to predictable interventions. Ongoing work is already validating this approach in pharmacological MRI, assessing the drug-target mechanisms behind CBF-based functional changes induced by selective serotonin reuptake inhibitors (SSRIs), and in clinical cohorts where a known deficit such as dopamine loss in Parkinson’s Disease likely affects the normal functioning of networks related to specific molecular pathways. Finally, a relatively short PLD (1.0 s) compared to the recommended PLD of 1.8 s for pCASL^16^ was employed for this study. Shorter PLDs can lead to intravascular artifacts if blood does not have adequate time to reach capillary-feeding small arteries. Despite this, this dataset has been used in another study to estimate and compare ASL- and BOLD-based resting state networks^24^, showing that they can be consistently detected using the CBF data. Other studies have also employed short PLDs^75^ and analyzed FC with a 3D pCASL sequence with PLD = 1.0 s and a BOLD EPI sequence^69^, finding robust ASL-based connectivity and considerable overlap between ASL and BOLD networks. However, future studies should specifically examine the effect of PLD on resting state FC.

### Conclusion

This study shows that the molecular enrichment of ASL fMRI timeseries is feasible and produces molecular-enriched FC maps that share features with those produced from BOLD fMRI, but also show particularities which origins we cannot ascertain yet but will need to be explored in future studies. Given the more direct link between ASL perfusion signal and neuronal activity, our integrative approach is likely to become a valuable asset in neuropharmacological studies investigating the effects of new or existent compounds on the brain or in clinical studies investigating functional alterations in brain disorders, particularly when the use of BOLD fMRI might fall short.

## Supplementary Information

Supplementary Figure 1.

## Data Availability

Data is available at https://openfmri.org/dataset/ds000216/.

## Code availability

The react-fmri package can be found at https://github.com/ottaviadipasquale/react-fmri.
