# Supplementary material for: Molecular-enriched functional connectivity in the human brain using multiband multi-echo simultaneous ASL/BOLD fMRI
Source: Sci Rep. 2023 Jul 20;13:11751. doi: 10.1038/s41598-023-38573-0 (PMC10359289; doi:10.1038/s41598-023-38573-0)
Supplement: Supplementary file 1 — Supplementary Figure 1. [file 41598_2023_38573_MOESM1_ESM.pdf]

Molecular-enriched functional connectivity in the human brain using multiband multi-echo simultaneous ASL/BOLD fMRI

Ottavia Dipasquale, Alexander Cohen, Daniel Martins, Fernando Zelaya, Federico Turkheimer, Mattia Veronese, Mitul Mehta, Steven CR Williams, Baolian Yang, Suchandrima Banerjee, Yang Wang

Supplementary material

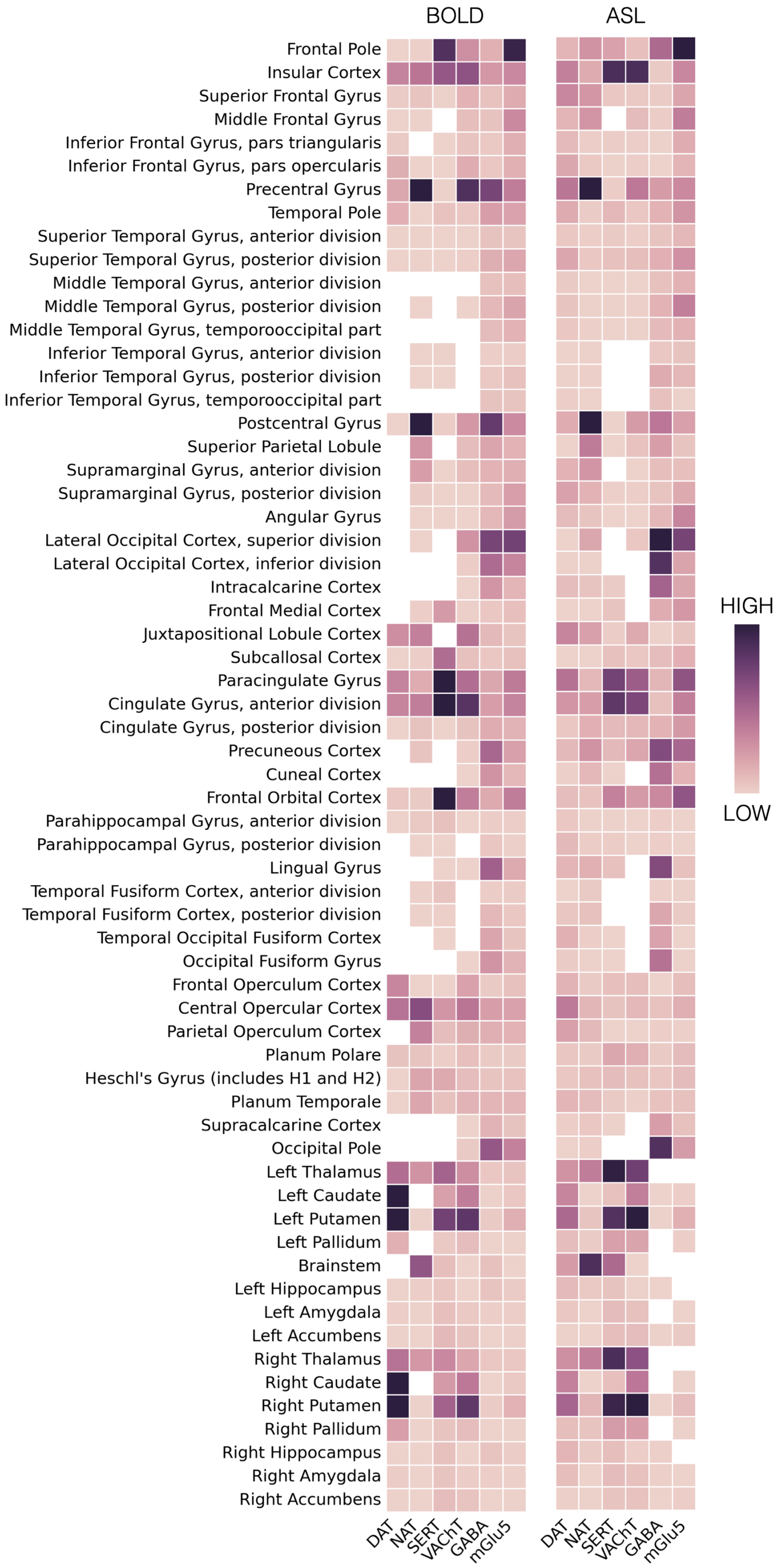

Supplementary Figure 1 **List of regions that reported significant within-group positive FC in the molecular-enriched functional maps derived from the BOLD and ASL datasets.** For each functional network and dataset, the color scale indicates the probability of the regions to belong to that network.
